# Oscillations and accelerations of ice crystal growth rates in microgravity in presence of antifreeze glycoprotein impurity in supercooled water

**DOI:** 10.1038/srep43157

**Published:** 2017-03-06

**Authors:** Yoshinori Furukawa, Ken Nagashima, Shun-ichi Nakatsubo, Izumi Yoshizaki, Haruka Tamaru, Taro Shimaoka, Takehiko Sone, Etsuro Yokoyama, Salvador Zepeda, Takanori Terasawa, Harutoshi Asakawa, Ken-ichiro Murata, Gen Sazaki

**Affiliations:** 1 Institute of Low Temperature Science, Hokkaido University, Kita-19 Nishi-8, Kita-ku, Sapporo 060-0819, Japan; 2 Japan Aerospace Exploration Agency, 2-1-1 Sengen, Tsukuba 305-8508, Japan; 3 Japan Space Forum, 3-2-1 Kandasurugadai, Chiyoda-ku, Tokyo 101-0062, Japan; 4 Japan Manned Space Systems Corporation, 2-1-6 Sengen, Tsukuba 305-0047, Japan; 5 Computer Centre, Gakushuin University, 1-5-1 Mejiro, Toshima-ku, Tokyo 171-0858, Japan

## Abstract

The free growth of ice crystals in supercooled bulk water containing an impurity of glycoprotein, a bio-macromolecule that functions as ‘antifreeze’ in living organisms in a subzero environment, was observed under microgravity conditions on the International Space Station. We observed the acceleration and oscillation of the normal growth rates as a result of the interfacial adsorption of these protein molecules, which is a newly discovered impurity effect for crystal growth. As the convection caused by gravity may mitigate or modify this effect, secure observations of this effect were first made possible by continuous measurements of normal growth rates under long-term microgravity condition realized only in the spacecraft. Our findings will lead to a better understanding of a novel kinetic process for growth oscillation in relation to growth promotion due to the adsorption of protein molecules and will shed light on the role that crystal growth kinetics has in the onset of the mysterious antifreeze effect in living organisms, namely, how this protein may prevent fish freezing.

The impurity effect for crystal growth in a bulk solution or melt is an important issue for understanding the crystal morphology and growth mechanism and a key technology for industrial crystallization. Most commonly, impurity molecules adsorbed on the growing interface work as pinning points of the growth steps, and the normal growth rates of the interfaces are strongly depressed. Generally, the growth rate of a crystal face should be constant with time when the driving force (such as supercooling or supersaturation) for growth is also constant. However, the possibility of periodic fluctuations in growth rates has long been discussed. Moreover, periodic fluctuations in growth rates are an intrinsic characteristic of an impurity effect and are associated with the periodic stripe patterns often observed in the cut sections of naturally or artificially grown crystals^1^,^2^,^3^. Although the mechanism of this oscillation depends on the adsorption-desorption kinetics of the impurity molecules, it has not been directly proven that oscillation in the normal growth rate occurs for any crystal. Because crystal growth experiments are typically performed on the ground, it is very difficult to escape from the unforeseen fluctuation of the growth rates generated by the natural convective flow around the growing crystal induced by gravity. Consequently, we were strongly motivated to measure the normal growth rates of crystal faces with a high degree of accuracy under microgravity conditions in which convective flow does not occur.

To accomplish this purpose, we studied ice crystal growth in supercooled water containing an antifreeze glycoprotein (AFGP) as an impurity (we call it an AFGP solution hereafter). Ice crystals grown from pure water at low supercooling conditions are disk shaped, with slowly grown basal faces and other rapidly grown faces^4^,^5^,^6^,^7^,^8^. However, ice crystals grown from a supercooled AFGP solution have a completely different morphology and growth kinetics^9^,^10^,^11^,^12^. We observed the free growth of ice crystals in supercooled AFGP solution under almost zero gravity (microgravity) conditions on the International Space Station (ISS), where convection is absent. There are several reasons why we used this system. First, it is well known that AFGP molecules, which are bio-macromolecules that function to prevent the freezing^9^,^11^,^13^,^14^,^15^ of living organisms in a subzero environment, adsorb on the growing interface of an ice crystal and strongly modify the growth morphology and kinetics. Consequently, AFGP molecules are the most effective impurities for ice crystal growth in supercooled water. Second, we had already successfully performed ice crystal growth experiments on the ISS in 2008–2009. Circular disk and dendrite morphologies with great hexagonal symmetries were obtained, and the pattern formation mechanisms of ice crystals were defined on the basis of a precise analysis of videos that were recorded on the ISS and downlinked to us^16^,^17^,^18^. Third, the system of ice crystal growth controlled by an AFGP can be regarded as a typical model for crystal growth controlled by bio-macromolecules, which is well known as biocrystallization^19^,^20^.

## Results

### Observation of ice crystal growth under microgravity

Microgravity experiments on the free growth of H_2_O ice crystals in supercooled water including an AFGP as an impurity were carried out using a newly developed space apparatus (Ice Crystal Cell 2) in the Japanese Experiment Module “KIBO” of the ISS during the period from November 2013 to June 2014. The AFGP concentration in water used for these experiments was fixed at 0.07 mg/mL, and the bulk supercooling (∆*T*_∞_) of the water at the beginning of the crystal growth was varied over the range between 0.1 and 0.5 K. The experiments were repeated 124 times for various supercoolings, and the growth of a polyhedral ice crystal surrounded by flat interfaces was always observed at the tip of a glass capillary inserted into the center of a growth cell filled with supercooled AFGP solution. The ice crystal growth was stably maintained in space over a period of 30 minutes (more than one hour in some cases) and observed using a Michelson interference microscope combined with a phase contrast microscope. However, an accident occurred in an operational system (Solution Crystallization Observation Facility) of ISS-KIBO shortly after the beginning of the experiments, and the function of the phase contrast microscope was completely lost. Coming out of this crisis, only the videos of the interference fringe images were acquired.

### Measurements of normal growth rates

**Figure 1:**
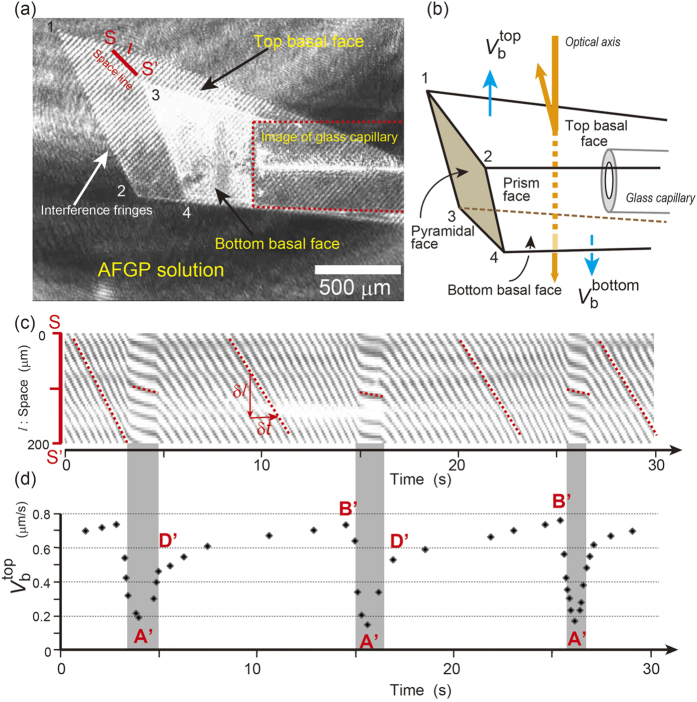
Interference fringes observed on the basal faces of ice that indicate the periodic fluctuation of the growth rate. (**a**) Snapshot of an ice crystal growing at the end of a capillary in the AFGP solution for ∆*T*_∞_ = 0.3 K. The corresponding video is provided as Supplementary Video 1. The region where the interference fringes appear corresponds to the basal face. The fringes observed clearly on the top basal face come from the interference between the reflection from that face and a reference mirror. The light region in Fig. 1(a), in which the images from the top and bottom basal faces overlap, is caused by the interference of the images from these two faces; in the video, that region actually oscillates in intensity. As the oscillation period depends on the increase in the crystal thickness during growth, we can separately determine the growth rates of the basal faces. (**b**) Illustration of the three-dimensional geometry for the ice crystal shown in (**a**). As the phase contrast images were accidentally lost, this illustration was determined from the arrangement of flat interfaces surrounding the external crystal shape based on the interference fringe images, in which only the top and bottom basal faces that orthogonally crossed the optical axis are visible. Numbers 1 to 4 indicate the corners of the crystal, and the places corresponding to the corners appear in the image in (**a)**. (**c**) Time-space image analyzed for the space line S to S’ indicated by a red line in (**a**). The normal growth rate, *V*, of this face is given by *V* = *βλ*/2*δn*_w_. Here, *β* is the migration velocity of the fringes that is given by the gradient (=*δl/δt*) of the line image of the time-space plot, *δ* the interval distance of the fringes, *λ* the wave length of the laser in air (670 nm) and *n*_w_ the refractive index of water (1.3328). (**d**) Growth rates at the center of the space line as a function of growth time. The maximum and minimum values of the growth rate for an oscillation period (B’ and A’, respectively) were determined from this analysis. Points A’, D’ and B’ correspond to the turning points for the kinetic process A’D’B’A’, as indicated in Fig. 3.

Interference images were obtained for 24 of the experiments and were analyzed to measure the growth rate as a function of the growth time. The typical procedure for the analysis of the interference fringe images is shown in Fig. 1. Figure 1(a) presents a snapshot taken from a video obtained in these experiments (Supplementary Video 1); it shows the movements of the interference fringes on the flat ice face at ∆*T*_∞_ of 0.3 K. We found that the straight fringes were arranged at regular intervals and laterally migrated with time but that their movement velocity periodically fluctuated. The directions of the interference fringes observed were constantly maintained over the lateral migration. Although we cannot provide any images to show the ice crystal geometry due to the accidental failure of the experimental apparatus, as described in the previous section, it is still possible to detect the geometry of the growing ice crystal from the images of the interference fringes. Figure 1(b) shows the three-dimensional geometry of the ice crystal shown in Fig. 1(a). Its geometry consists of two flat interfaces perpendicular to the optical axis and three trapezoidal flat interfaces connecting those flat interfaces. From the geometric arrangement of the glass capillary and growing ice crystal, we conclude that the flat interfaces with interference fringes should be the basal faces (namely, the top and bottom basal faces in Fig. 1(b)) and the faces connecting the basal faces should be one prism face and two pyramidal faces.

As the lateral movement of the fringes corresponds to the normal movement of the observed interface, the growth rates of the basal faces were obtained by analyzing a spatiotemporal image of a video. For example, to analyze the video shown in SI, we take the line *l* (S to S’) as shown in Fig. 1(a), which is referred to as the “space line”. Intensity profiles along this line are obtained for each frame of the video and plotted as a function of time. Then, a time-space image, as shown in Fig. 1(c), is finally obtained. The line images in this figure indicate the lateral movement of the interference fringes, and the gradients of these lines correspond to the normal growth rate of the top basal face (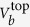), as shown by the dotted lines in the figure. 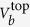 at the center position of the space line *l* versus the growth time plotted in Fig. 1(d) shows the periodic oscillation. The minimum and maximum values of the growth rates varied by a factor of more than five (0.13 to 0.73 μm/s in this case), and the oscillation period, which was approximately 11 s in this case, gradually become shorter with the increase in ∆*T*_∞_. The patterns of variation in 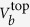 in the cycles were similar; that is, the growth rate gradually increased and then rapidly decreased immediately after it reached a maximum value. The amount of growth per cycle was approximately 6.33 μm in this case, and the average growth rate estimated by dividing the amount of growth by the cycle time was 0.58 μm/s.

A change in the period of the brightness was also observed in the central area of the top basal face. As the dark or bright images occur depending on the phase differences between the light beams reflected from both the top and bottom basal faces, the change in the period of the brightness is proportional to the rate of increase of the crystal thickness (namely, the distance between the top and bottom basal faces). Consequently, the growth rate of the bottom basal face (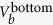) can be independently determined by combining the measurements of 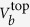 and the brightness change period. A detailed explanation and confirmation of the determination of 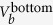 are given in Supplementary Figure 1.

### Oscillatory growth observed on the basal faces

**Figure 2:**
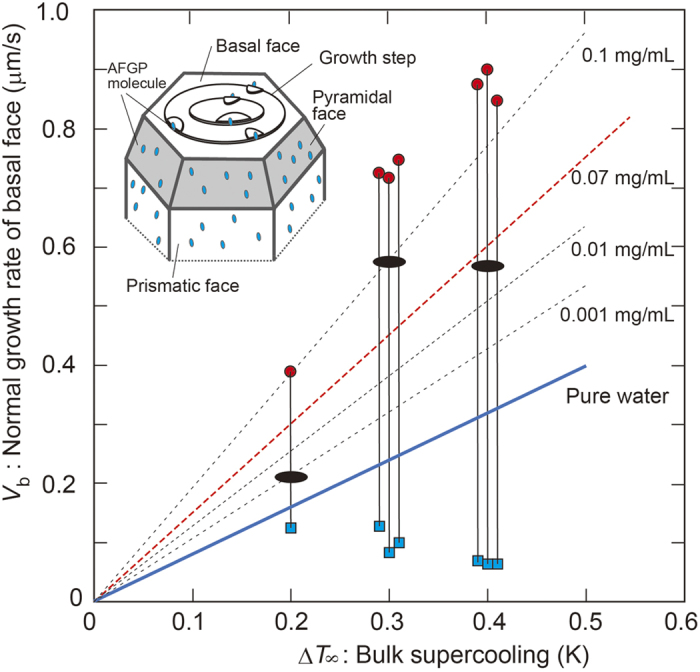
Growth rates obtained under microgravity conditions on the ISS as a function of the Δ*T*_*∞*_ of the AFGP solution. Red solid circles and blue solid squares indicate the maximum and minimum values of the growth rates during the periodic fluctuations of growth, respectively, and the solid lines connect the corresponding data points. Ellipsoidal points indicate the growth rates averaged for a long stretch of time (more than 10 minutes). As a reference, the dotted lines indicate the growth rates along the c-axes of ice crystals for various AFGP concentrations, based on results of ground experiments (see Supplementary Figure 2). Data obtained at ∆*T*_∞_ = 0.3 K and 0.4 K are plotted with slight shifts to prevent overlap. At ∆*T*_∞_ = 0.1 K, no ice crystal was obtained because the growth rate was too small, and at a supercooling of 0.5 K or more, interference fringes could not be obtained because the growth was too fast. The maximum values of the growth rates increased with the increasing ∆*T*_∞_, whereas the minimum values of the growth rates showed the opposite trend. The illustration inside the figure shows the anisotropic adsorption states of the AFGP molecules between the basal and other (prismatic and pyramidal) faces. The AFGP molecules are preferentially adsorbed on the prismatic and pyramidal faces of the ice crystals and prevent the growth of these faces due to the pinning effect. By contrast, if the AFGP molecules are preferentially adsorbed on the prismatic planes that construct the step edges on the basal faces, they can work as new sources of growth steps and are involved in the growth promotion of the basal faces. Consequently, the preferential adsorption of AFGP molecules on the prismatic and pyramidal faces might produce the opposite effects of the inhibition and promotion of growth.

Periodic oscillations in the growth rate were clearly detected for all of the basal faces when interference fringes appeared, regardless of ∆*T*_∞_. Figure 2 shows the ∆*T*_∞_ dependence for the maximum and minimum values of the growth rate during the periodic oscillations (red circles and blue squares, respectively, with the corresponding points for each experiment connected by solid lines). The average growth rates were also plotted using black solid circles. The difference between the maximum and minimum values, namely, the amplitude of the growth rate oscillation, became larger with increasing ∆*T*_∞_.

The minimum values of the growth rates obtained in space were always smaller than those obtained for pure water (blue solid line). This trend is related to the generally known effect of convection on crystal growth, namely, the growth rate will decrease under microgravity conditions due to the primary extensity of a diffusion field around the growing crystal. By contrast, the maximum values of the growth rates obtained in space were larger than those obtained for a 0.07 mg/mL AFGP solution (red broken line). This trend is also related to the effect of the AFGP concentration increasing at the vicinity of the growing interface due to the rejection of AFGP molecules. It should be noted that the average values of growth rates during a cycle correspond to the growth rates along the c-axis at an AFGP concentration of 0.07 mg/mL that were obtained in ground experiments.

We should emphasize that growth oscillation was clearly observed only after we measured the growth rate under long-term microgravity conditions. Indeed, in experiments of the free growth of an ice crystal in an AFGP solution carried out on the ground, the periodic fluctuation of the growth rates was not observed for the basal faces or other crystalline faces. Some experiments on ice crystal growth using Ice Crystal Cell 2 were also carried out on the ground during the last minute of the launch. Although the irregular fluctuation of the interference fringe migration was observed, no periodicity was detected. We should emphasize that a unique observation of the periodic growth of an ice crystal was reported, in which one-directional growth of the prism faces of ice crystals was observed^21^. However, as the ice crystal growth in this growth system was carried out in a thin rectangular growth cell of 50-μm thickness maintained in a horizontal position, the convection flow effect in front of the growing interface might have been suppressed^22^,^23^. Consequently, the periodicity of the growth rate variation is an innate characteristic of the ice crystal growth affected by an AFGP and is suppressed by the convective flow caused by the gravity effect.

### Growth promotion of the basal faces of ice in AFGP solution

The straight lines in Fig. 2 show the average growth rates along the c-axis obtained in pure water (blue solid line) and in solutions with various AFGP concentrations (black broken lines), which were measured in separate experiments carried out under the effect of convection flow on the ground. The ∆*T*_∞_ dependence of the growth rate at the AFGP concentration of 0.07 mg/mL, which was presumed from the concentration dependence of the growth rate, is indicated by a red broken line in this figure (see Supplementary Figure 1 for details). It should be noted that as these growth rates were obtained from the time variations of the growth morphologies, they were regarded as average growth rates, and oscillatory variations of the growth rates were never seen. These results indicate an important feature of AFGP molecules, in that they greatly increase the growth rate of the basal faces of ice crystals. As the growth promotion of the basal faces was observed regardless of the gravity levels, we emphasize that the origin of growth promotion is not due to the effect of microgravity.

A comparison of the experimental results obtained on the ground and in space suggests that the AFGP molecules adsorbed on the basal faces may work as sources of growth promotion (namely, adsorption-promotion effect) and not as sources of growth inhibition (namely, adsorption-inhibition effect), which is completely opposite to the previously believed effect of adsorbed impurity molecules on the growing interface. That is, growth depression due to the poisoning of kink sites or the pinning of step advancement at the points of adsorption on the crystal interface^20^.

## Discussion

### Mechanism of growth promotion of the basal faces of ice by adsorbed AFGP

We here attempt to present a schematic explanation of the new findings. An ice crystal growing in pure water takes on a circular disk shape surrounded by two flat basal faces and one rounded edge face^4^,^8^,^24^,^25^. This observation indicates that the basal faces grow by a layer-by-layer mechanism but that the growth of the edge face is controlled at a rate at which the latent heat released at the edge is removed by diffusion^24^,^25^. By contrast, the ice morphology in an AFGP solution changes to a polyhedral shape surrounded by two parallel hexagonal faces (basal faces), six prismatic faces and trapezoidal pyramidal faces connecting the basal and prismatic faces^15^,^26^,^27^ (see the illustration in Fig. 2).

Previously, AFGP molecules were thought to be adsorbed on the prismatic or pyramidal faces but not on the basal faces and to have the ability to smooth the rough interfaces^15^,^27^,^28^,^29^, which is recognized as a “smoothing transition”, in contrast to the well-known effect of the roughening transition by an impurity^30^,^31^. Moreover, in previous experiments^27^ and simulations^28^, no adsorbed AFGP molecules were seen on the basal faces. However, we found that the growth of the basal faces was strongly accelerated by the presence of the AFGP molecules. What causes this drastic change?

It is well known that the basal faces of ice crystals grow by a layer-by-layer mechanism even in pure water. This fact prompted us to speculate that the AFGP molecules are adsorbed on the edges of the growth steps on the basal face because those should be constituted by faces that include the prismatic or pyramidal faces, as shown by the illustration in Fig. 2. As the average size of the AFGP molecules (approximately 3 nm in diameter^32^) is much larger than the height of the elementary step (0.37 nm) on the basal face^33^, the adsorbed molecules would protrude from the growth steps and may work as sources of growth steps for the basal face. This process may cause the adsorption-promotion effect of the impurity molecules for crystal growth.

### Processes triggering growth oscillation

**Figure 3:**
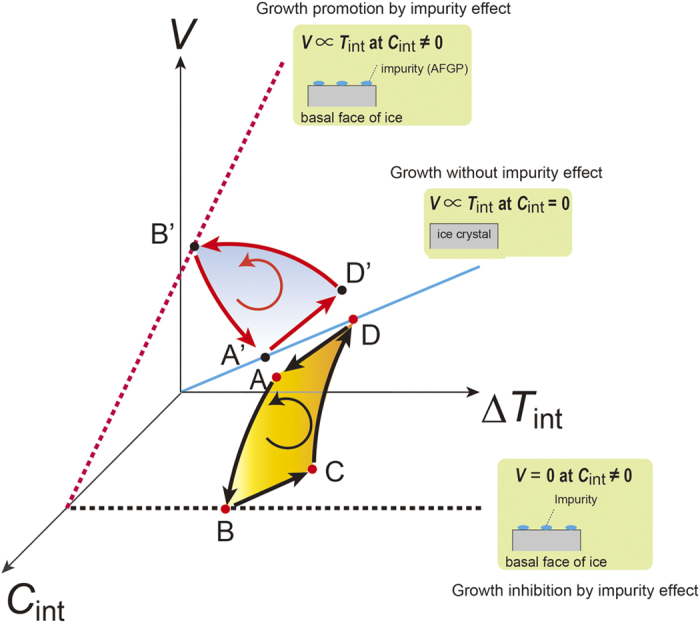
Schematic illustration of growth kinetic processes to explain the origin of the growth oscillation. The solid blue line indicates the interfacial supercooling dependence of the growth rate for the face without any impurity molecules, and the black and red broken lines indicate the same for the faces with adsorbed impurity (AFGP) molecules. A previously known kinetic process (ABCDA) for growth oscillation based on the effect of AFGP molecules, as the growth rate decreases due to step pinning at the adsorption sites, is shown by the continuous process formed by black solid arrows. Meanwhile, a new kinetic process (A’D’B’A’) for growth oscillation based on the adsorption-promotion effect of impurity molecules, as the growth rate increases due to the enhancement of the nucleation ability of the growth steps on the basal face, is shown by the continuous process formed by red solid arrows.

Now let us consider the origin of the periodic oscillation of growth rates induced by the adsorption effect of the impurity molecules. Figure 3 schematically illustrates the growth rate (*V*) changes based on the kinetic processes that are expected to occur on growing interfaces under the effect of impurity molecules. The kinetic processes originate from the interactive variation between interfacial supercooling (∆*T*_int_) and the concentration of adsorbed impurity molecules at the interface (*C*_int_). First, we consider previously discussed models for growth oscillations^34^,^35^,^36^,^37^,^38^, according to which there is strong growth depression due to the adsorption of impurity molecules at the interface. That is, growth is stopped when *C*_int_ becomes greater than a critical value, as shown by a black dotted line in Fig. 3. Assuming that the growth rate at *C*_int_ = 0 is proportional to ∆*T*_int_ (blue solid line), growth oscillation is expected to occur due to the sequential kinetic process, shown as the loop (ABCDA) indicated by black arrows in Fig. 3. That is, (1) A to B: *V* is depressed due to an increase in *C*_int_; (2) B to C: *V *= 0, but *C*_int_ decreases due to the desorption of impurity molecules and Δ*T*_int_ increases at the same instance because of a decrease in the latent heat released at the interface; (3) C to D: *V* is accelerated when *C*_int_ reaches a critical value; and then (4) D to A: *V* gradually decreases with the increase in *C*_int_ due to the expulsion of impurity molecules at the growing face, known as “impurity rejection”, and Δ*T*_int_ decreases again with the increasing latent heat release. This scenario has been universally recognized as a model for growth oscillation induced by an impurity.

However, this model is not applicable to the growth oscillation observed on the basal faces in our experiment, because the model is related to the adsorption-inhibition effect of impurity molecules. The red dotted line in Fig. 3 indicates that the gradient of *V* versus Δ*T*_int_ becomes steeper with the increasing *C*_int_ compared to the Δ*T*_int_ dependence of *V* at *C*_int_ = 0, as shown by the straight blue line. Based on this adsorption-promotion effect, we propose a completely new process (A’D’B’A’) as the origin of the growth oscillation, which is shown by red arrows in Fig. 3. That is, (1) B’ to A’: *V* is abruptly depressed by the disappearance of adsorbed molecules due to an as yet unknown reason; (2) A’ to D’: *V* gradually increases because Δ*T*_int_ increases with the reduction of the latent heat release at *C*_int_ ≈ 0, and then *C*_int_ is simultaneously increased by the rejection of AFGP molecules at the growing interface; and (3) finally, D’ to B’: *V* increases further with the increasing *C*_int_, but Δ*T*_int_ starts to decrease with the further increase in the latent heat release at the same time. Although we have not yet provided any direct experimental evidence or mathematical model, it is important to note the possibility of a new impurity effect for crystal growth because nobody expected the occurrence of this oscillatory phenomenon in crystal growth before our findings in space.

Finally, we consider how this new scenario corresponds to the actual variations in the growth rates observed on the basal faces of ice. The points that appeared in the new kinetic process shown in Fig. 3 are indicated as the corresponding points of B’, A’, and D’ in Fig. 1(d). The most important process that drives this oscillatory growth is the sudden depression of the growth rate labeled by B’ to A’, which corresponds to the abrupt drop-off in *C*_int_. Let us return to Supplementary Video 1. We observe that a sudden termination of the lateral movement of the interference fringes occurs with the propagation of the sinuate variation observed on the fringes, which may occur by a macro step. The conventional model for the impurity effect of crystal growth assumes that the impurity molecules adsorbed on the growing faces can be covered by the macrosteps. The same process may occur even for the crystal growth of the basal face adsorbed by AFGP molecules, but the difference between them is that growth is promoted at the former but reduced at the latter.

### Freezing inhibition of living organisms by AFGP

**Figure 4:**
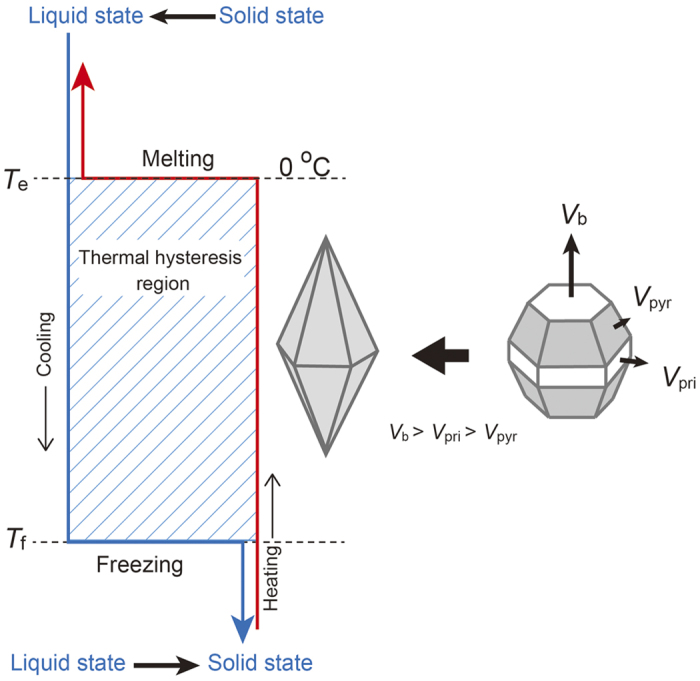
Schematic illustration to explain the establishment of freezing inhibition by AFGP molecules in relation to the growth promotion of the basal face. The blue and red solid lines in the left illustration indicate the cooling and heating processes of the AFGP solution, respectively. An AFGP solution never freezes in the temperature range between the equilibrium melting point, *T*_*e*_, and the freezing temperature, *T*_*f*_. This temperature range is defined as the thermal hysteresis. In this temperature region, many ice particles with a dodecahedral shape, as shown at the center of this figure, are actually observed in the blood of living organisms. When the blood temperature reaches the freezing temperature, the blood suddenly starts to freeze. It is important to note that the dodecahedral shapes are formed on the basis of the well-known principle of the growth of a polyhedral crystal.

Finally, it should be noted that the growth promotion of the basal face is essential to fulfilling the freezing inhibition function for living organisms under subzero conditions. Figure 4 presents a schematic explanation of freezing inhibition. When water containing an AFGP is gradually cooled down, it never freezes at the equilibrium melting point of ice (*T*_*e*_) but rather at a critical temperature (*T*_*f*_), as shown by the blue line in Fig. 4. The melting of ice crystals during the heating process (the red line in Fig. 4), however, can occur at the bulk melting point (*T*_*e*_). The difference between *T*_*e*_ and *T*_*f*_ is defined as a thermal hysteresis and is dependent on the AFGP concentration^11^. Many tiny ice particles with a dodecahedral shape (several tens of micrometers in size) surrounded by only pyramidal faces (as shown at the center in Fig. 4) are observed in the blood of living fishes in this thermal hysteresis region^39^,^40^. These ice particles do not continue to grow in this region but start to grow exponentially at the instant when the serum temperature drops below *T*_*f*_ 41. The reason why the dodecahedral ice particles can stably exist in serum is explained by a key principle for the growth of polyhedral crystals, namely, “the flat faces with faster growth rates are truncated by the faces with slower growth rates and finally the polyhedral crystal is surrounded by only flat faces with the lowest growth rate”. Consequently, the formation of dodecahedral ice particles suggests that the growth rates of the individual faces must maintain the relationship of *V*_*b*_>*V*_pri_>*V*_pyr_, where *V*_*b*_, *V*_pri_, and *V*_pyr_ are the growth rates for the basal, prismatic and pyramidal faces, respectively (illustration on the right in Fig. 4). When all of the basal and prismatic faces on an ice crystal disappear, the ice crystals should be surrounded by only pyramidal faces, which have the slowest growth rate, and finally the formation of a dodecahedral ice particle is complete. This external form of an ice crystal prevents its further growth, and the water is thus kept in a supercooled state. In conclusion, the prevention of freezing in living organisms cannot be solely explained by the growth depression effect of AFGP molecules on ice crystals. In other words, the anisotropic functions of AFGP molecules for ice crystal growth are essential for the prevention of freezing of living organisms.

Our findings in space indicate not only an important concept for understanding the mechanism of the antifreeze function of an AFGP but also a possible mechanism for the formation of the striped pattern generally observed inside mineral crystals. The ice crystal growth process in an AFGP solution is the same as that for inorganic crystal growth controlled by biological macromolecules, namely, a biocrystallization process. An understanding of the mechanism of this oscillatory growth may provide new insight into exploring crystalline material formation within an organism, and it will also provide a new target for the mathematical analysis of general oscillatory phenomena.

## Methods

### Ice crystal growth and imaging

The experimental apparatus used on the ISS was designed for observations of ice crystal growth in water including an AFGP, and it was named Ice Crystal Cell 2 (ICC2), as it was developed for the second project of ice growth experiments on the ISS following the first project^16^,^17^,^18^ carried out in 2008–2009. ICC2 was composed of two parts: an ice growth apparatus and an optical system. The main parts of the ice growth apparatus were a spherical growth cell (SGC) with an inside diameter of 40 mm equipped with two opposed observation windows (the inside volume of the SGC was approximately 30 mL) and an ice nucleation unit (INU) that incorporated a thin glass capillary (0.6 mm in outer diameter and 60 mm in length) to enhance the formation of a seed crystal of ice by rapid cooling. The outer end of the capillary was inserted into the center of the SGC to initiate the free growth of a single ice crystal in the SGC. The INU was also retrofitted to rotate the capillary around its central axis within an angular range of ±45°, a critical improvement to adjust the inclination of the ice/water interface in a direction perpendicular to the optical axis. The AFGP solution in the SGC was cooled down by Peltier cooling elements on the outside wall surface, and the solution temperature was controlled within an accuracy of ±0.05 K using a PID controller. The capillary end inside the INU was able to be rapidly cooled down to nucleate ice crystals using small Peltier elements.

An ice crystal growing in the SGC was imaged *in situ* using the optical system installed in ICC2, which was a Michelson-type interference microscope combined with a phase contrast microscope. As the reflection coefficient of light at the ice/water interface is extremely low (approximately 0.00014, one five-hundredth of that of a glass surface), nobody has succeeded in obtaining the interference fringes with visible contrast until now. However, by adjusting the intensity of both the reflected light beams from the ice/water interface and those from a reference mirror using a specially designed optical system, and then taking advantage of the polarization of the light beams, we were able to obtain interference fringes with adequate contrast, which appear as shown in Fig. 1(a). The rotation of the glass capillary around its central axis helped to adjust the direction of the basal face to the optical axis of the interference microscope. A phase contrast microscope was included in this optical system to obtain clear images of the growth steps migrating on the ice/water interface.

### Materials

Antifreeze proteins (AFPs) and antifreeze glycoproteins (AFGPs) are well-known functional bio-macromolecules that help fish^11^, insects^42^,^43^, plants^44^, and bacteria^45^ to survive when exposed to subzero environments. These proteins drastically modify the growth morphologies^9^,^14^ and inhibit the recrystallization^12^ and growth^15^ of ice crystals. These molecules have drawn considerable interest due to their applicability in the frozen food industry^46^ and in medical treatments^41^,^47^ (for example, as a cryosurgery adjuvant, in blood preservation, and even the possibility of human organ preservation). In our space experiments, we used an AFGP collected from *Gadus ogac* plasma to make a sample solution. The AFGP was supplied by A/F Protein Inc., Waltham, MA, USA. The peptide backbone of each AFGP molecule consists of repeating alanine-alanine-threonine (*Ala-Ala-Thr*) tripeptide units^6^. The *Thr* peptide has a disaccharide residue (3-*O*-(*β*-_D_-galactosyl)-_D_-*N*-acetylgalactosamine), where the two sugar groups are linked as a glycoside to the hydroxyl oxygen of the threonine residue. Eight distinct fractions of AFGPs can be isolated, with most of the differences being in the number of tripeptide repeats, which range from 50 to 4 for bands 1–8. The molecular weights range from 32 to 2.7 kDa. The AFGP7–8 used in the present experiments is a mix of naturally occurring smaller glycopeptides (molecular mass < 4 kDa). The AFGP was dissolved in degassed pure water, and the AFGP concentration of the solution used in space was adjusted to 0.07 mg/mL based on a colorimetric analysis of the solution.

The secondary structure of an AFGP molecule has been shown by precise analysis using various methods to be a highly flexible extended helix^48^. By contrast, Uda *et al*.^49^ showed using ATR-FTIR spectroscopy that the AFGP molecules adsorbed on the ice–water interface undergo a conformational change from a flexible extended helix to an α-helix. Molecular dynamics simulation^28^ also indicated that molecules with an α-helix conformation can be absorbed selectively on the prismatic or pyramidal faces of ice crystals.

### Procedures for space experiments on ISS-KIBO.

Before launching, the interior portions of the SGC and INU were filled as much as possible with the AFGP solution, taking care not to create any solution-vapor surface. It was very important to prevent Marangoni convection, which might be the preferred source of liquid flow in microgravity. Then, ICC2 was loaded in the Japanese unmanned cargo transporter, the H-II Transfer Vehicle “KOUNOTORI 4 (HTV4)” and launched toward the ISS in August 2013 from the Tanegashima Space Center of JAXA. After arriving at ISS-KIBO, ICC2 was placed on the stage of the Solution Crystallization Observation Facility (SCOF), which had been originally installed as a common facility in KIBO, and the experiments were carried out in the period from December 2013 to June 2014. An experiment proceeded simply by separately changing the preset temperatures of the SGC and INU by remote-controlled commands sent from the ground. The procedure of an experiment started by creating a supercooled solution of uniform temperature in the SGC, and then the outer end of a glass capillary end inside the INU was rapidly cooled to initiate ice nucleation. The nucleated ice particles continued to grow inside the capillary, and only one ice particle ultimately survived the competitive growth of adjacent ice particles when the freezing front in the capillary reached the inner end. As an ice crystal with its c-axis perpendicular to the capillary axis could definitely survive^50^, an ice crystal that started to grow at the end of the capillary will grow with its c-axis always perpendicular to the central axis of the capillary. After the free growth of an ice crystal started, its c-axis direction was adjusted to the optical axis by rotating the glass capillary. When the reflected light beam from the ice basal face became exactly parallel to the optical axis, interference fringes suddenly appeared on the basal face. The oscillatory growth continued for more than 30 min (with smaller ∆*T*_∞_, more than 60 min) after the appearance of interference fringes. Both video images and temperature data were downlinked from the ISS to the ground with a time lag of only a few seconds and were recorded as experimental data. Growth was terminated at an appropriate time by a shutdown of the electric power supply to prevent an excessive increase in the internal pressure of the SGC. After melting the whole crystals in the SGC and INU, the growth apparatus was returned to its initial state. In total, 124 experiments were carried out under various ∆*T*_∞_ conditions. Although ice crystal growth was successfully observed without any exceptions above *∆T*_∞_ = 0.2 K, interference fringes were observed on the basal faces in only 24 experiments. However, considering the difficulty in the distance control of space-borne experiments, this success probability (20%) is sufficiently high.

We experienced an accident in a power supply unit in the operational system (SCOF) of ISS-KIBO shortly after beginning the experiments. Unfortunately, the impact of this failure spread to the optical system of the phase contrast microscope in ICC2 in the process of mending this failure, and the function of the phase contrast microscope was lost. It was impossible to repair the failure that occurred in ICC2. As a result, the phase contrast images for ice crystal growth were completely lost in the actual space experiments, and we had to analyze the experimental data using only the interference fringe images of ice crystal growth. This accident caused some problems in the analysis of the experimental data, namely, on the external forms of the growing ice crystals and the images of the growth steps on the interfaces.

## Additional Information

**How to cite this article:** Furukawa, Y. *et al*. Oscillations and accelerations of ice crystal growth rates in microgravity in presence of antifreeze glycoprotein impurity in supercooled water. *Sci. Rep.* **7**, 43157; doi: 10.1038/srep43157 (2017).

**Publisher's note:** Springer Nature remains neutral with regard to jurisdictional claims in published maps and institutional affiliations.

## Supplementary Material

Supplementary Video 1

Supplementary Video 2

Supplementary Information
