# Supplementary material for: Oscillations and accelerations of ice crystal growth rates in microgravity in presence of antifreeze glycoprotein impurity in supercooled water
Source: Sci Rep. 2017 Mar 6;7:43157. doi: 10.1038/srep43157 (PMC5338005; doi:10.1038/srep43157)
Supplement: Supplementary Information [file srep43157-s3.pdf]

Supplementary Information

Oscillations and accelerations of ice crystal growth rates in microgravity in presence of antifreeze glycoprotein impurity in supercooled water

Yoshinori Furukawa[1*], Ken Nagashima[1], Shun-ichi Nakatsubo[1], Izumi Yoshizaki[2], Haruka Tamaru[2], Taro Shimaoka[3], Takehiko Sone[4], Etsuro Yokoyama[5], Salvador Zepeda[1†], Takanori Terasawa[1], Harutoshi Asakawa[1‡], Ken-ichiro Murata[1] & Gen Sazaki[1]

[1]Institute of Low Temperature Science, Hokkaido University, Kita-19 Nishi-8, Kita-ku, Sapporo 060-0819, Japan

[2]Japan Aerospace Exploration Agency, 2-1-1 Sengen, Tsukuba 305-8508, Japan

[3]Japan Space Forum, 3-2-1 Kandasurugadai, Chiyoda-ku, Tokyo 101-0062, Japan

[4]Japan Manned Space Systems Corporation, 2-1-6 Sengen, Tsukuba 305-0047, Japan

[5]Computer Centre, Gakushuin University, 1-5-1 Mejiro, Toshima-ku, Tokyo 171-0858, Japan

[*] Corresponding author, frkw@lowtem.hokudai.ac.jp

[†] Present address, CCZ Crystal R&D, St. Peters, MO 63376, USA

[‡] Present address, Graduate School of Sciences and Technology for Innovation, Yamaguchi University, 2-16-1 Tokiwadai, Ube 755-8611, Japan

Supplementary Figure 1. Confirmation of the measurements of growth rates for top and bottom basal faces.

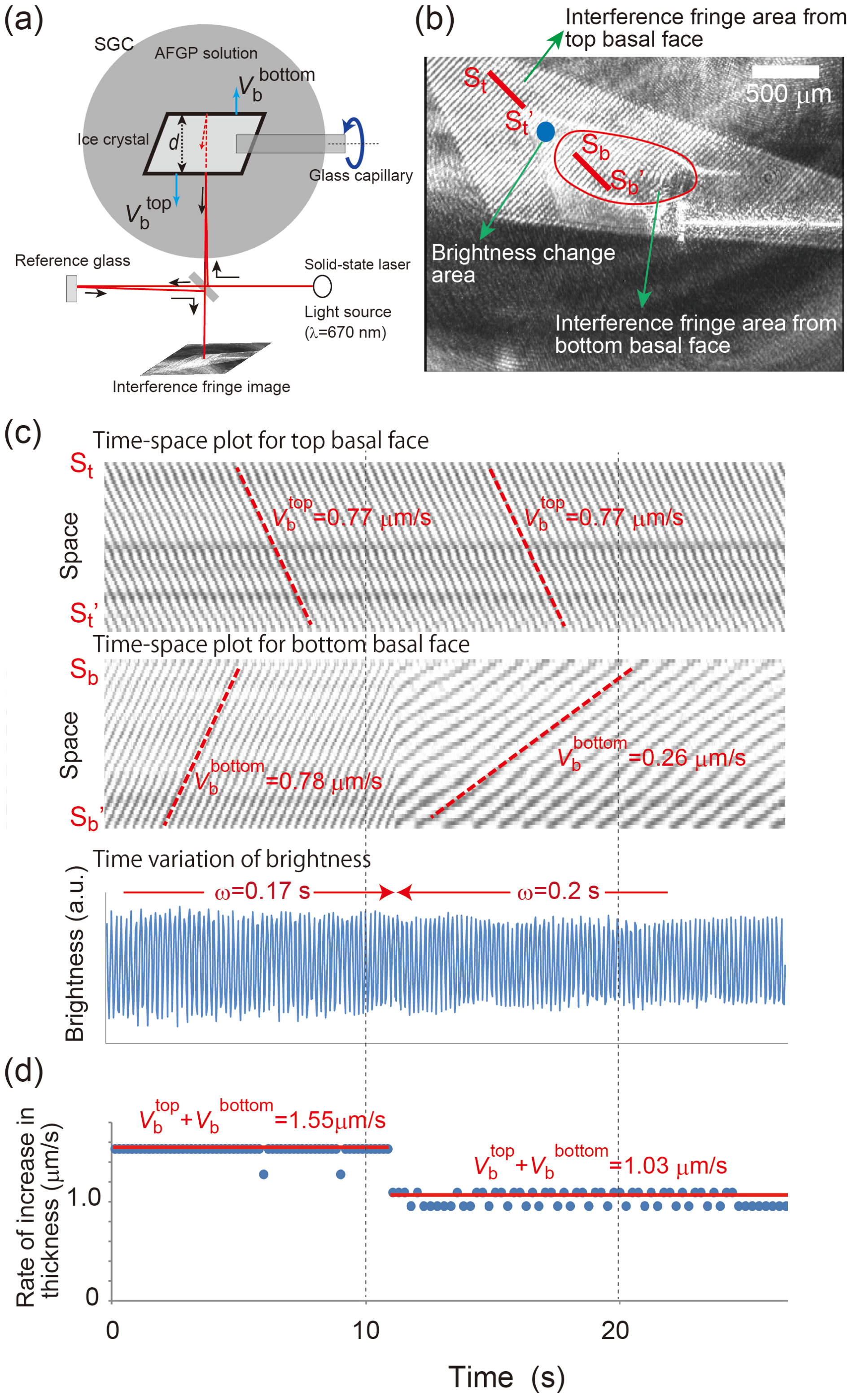

(a) Schematic illustration of the optical system. Interference fringes may occur between the light beams reflected from the reference glass surface and the top basal face, and V_b^{top} can be measured precisely as shown in Figure 1. The change in the period of the brightness in a particular region in the video images was also observed. We presumed that the interference between the light beams reflected from the top and bottom basal faces gives rise to this phenomenon and that its period is proportional to the rate of the increase in thickness of an ice crystal. (b) Snapshot of a video, provided as Supplementary Video 2. In this video, we can observe both the interference fringes coming from the top basal face and the area with a brightness change at the center of the ice basal face. Interference fringes moving in the opposite direction are observed in the area with a brightness change. These interference fringes came from the interference between the light beams reflected from the bottom basal face and the surface of the reference glass, and V_b^{bottom} can be separately determined from the analysis of these fringes. By contrast, the change in the period of brightness observed in the central area of the top basal face occurs by the interference between the light beams reflected from the top and bottom basal faces, and the period of the brightness change is proportional to the rate of the increase in crystal thickness. (c) Independent measurements of V_b^{top}, V_b^{bottom} and the rate of increase in thickness. The first and second figures show the time-space images analyzed for the space lines S_t to S_t' and S_b to S_b', indicated by red lines in (b), respectively. V_b^{top} and V_b^{bottom} can be independently determined. The third figure shows the time variation of the brightness averaged in the blue circular area indicated in (b), and the period of the brightness oscillation, ω, can be measured. Then, the rates of increase in thickness are determined by the equation $\delta d/\delta t = \lambda/2\omega n_i$, where d is the thickness of the ice crystal and n_i the refractive index of ice (1.3078). (d) Confirmation of the measurements of the growth rates. We confirmed that the values of $V_b^{top} + V_b^{bottom}$ estimated from the two growth rates separately determined from a time-space plot analysis of the interference fringes (blue solid circles) correspond completely to the rates of increase in thickness (red lines) determined from the brightness change.

Supplementary Figure 2. Growth rate along the c-axis as a function of ΔT_∞ measured on the ground.

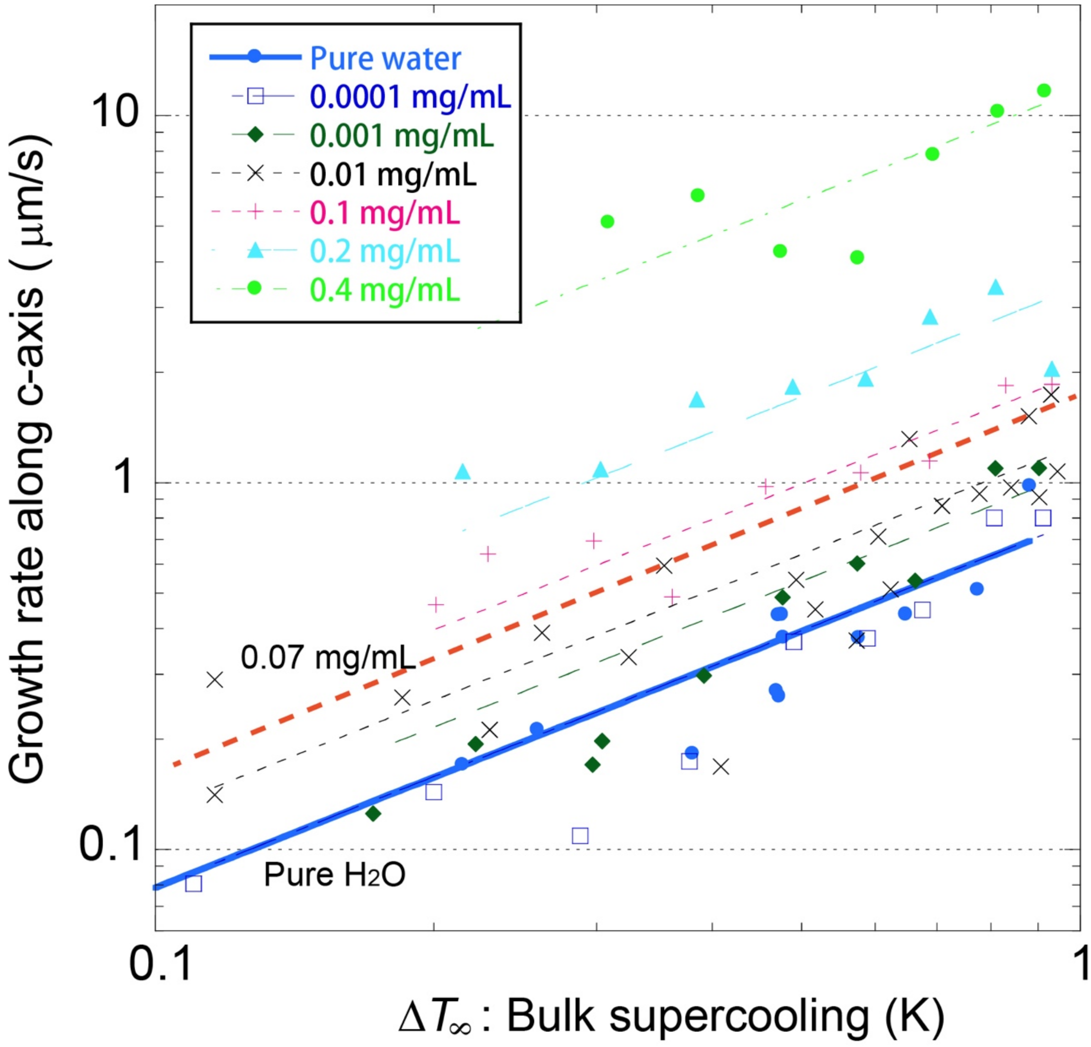

A single ice crystal was grown in a small cylindrical glass cell (20 mm in diameter and 20 mm in length) that was put in a circulating constant-temperature bath. The growth rates along the c-axis were measured by different methods depending on the growth form, namely, the use of a Mach-Zehnder interferometer for thinner ice crystals and the use of a conventional microscope for the thickness measurements of thicker ice crystals. As the level of accuracy of the measurements was much lower than that for the measurements carried out in the space experiments, we could not detect any oscillatory behavior. By analyzing the ΔT_∞ dependence of the growth rates, we confirmed that the growth rates were proportional to ΔT_∞ for all AFGP concentrations. By using the

concentration dependence on the growth rate, we determined the relation between the growth rate and ΔT_∞ for an AFGP concentration of 0.07 mg/mL, which is shown by the red broken line in the figure. These lines are reflected to the dotted and red lines in Figure 2.

Supplementary Video 1. Interference fringe migration observed on the ice basal face in space.

Equally spaced interference fringes appear on the growing basal face of ice and migrate in the direction from the upper left to the lower right. The migration velocity suddenly decreases at the same time as the macro step sweeps across the basal face, and then the interference fringes gradually gain speed. This process is repeated with a constant period. The change in the period of the brightness was also observed in the central light region of the basal face in Fig. 1(a). ΔT_∞ at the start of the ice growth was 0.3 K. This video is 30 sec in time length, and it was cut from the original much longer video (approximately 60 min) obtained on the ISS. No image processing was performed on this video, and it reflects a real-time video reproduction. The width and height of the image are 2.4 mm and 1.6 mm (640x480 pixels), respectively.

Supplementary Video 2. Interference fringe migration and periodic change of brightness observed on the ice basal face in space.

Conjunction of three events observed on the ice basal face in space, namely, interference fringe migration related to the growth of the top basal face (peripheral area), interference fringe migration related to growth of the bottom basal face (central area), and the brightness change related to the interference between the reflected light beams from both basal faces was observed. Note that the migration directions are opposite in the peripheral and central areas. ΔT_∞ at the start of the ice growth was 0.3 K. This video is 25 sec in length, and it was cut from the same video as was Supplementary Video 1. No image processing was performed on this video, and it reflects a real-time video reproduction. The width and height of the image are 2.4 mm and 1.6 mm (640x480 pixels), respectively.
